# Connecting with community in a public health emergency: a pilot project example for local public health and the communities they serve

**DOI:** 10.1007/s10389-025-02615-w

**Published:** 2025-11-07

**Authors:** Janice Valenzuela, Melissa Marver, Lakeeta Watts, Crystal Peterson, Chris Frakes, Jill Denson, Kate H. Gillespie

**Affiliations:** 1 The University of Wisconsin Prevention Research Center, Madison, WI, USA; 2 The University of Wisconsin, Madison, WI, USA; 3 Essentially Empowered, Inc., Milwaukee, WI, USA; 4 Southwestern Wisconsin Community Action Program, Dodgeville, WI, USA; 5 The University of Wisconsin School of Nursing, Madison, WI, USA

**Keywords:** Community health workers, Emergency response, Pandemic, COVID-19, Culturally appropriate, Health equity

## Abstract

**Background:**

The extensive infrastructure of community-based programs serving pregnant and postpartum women and their families could be engaged in a scalable and sustainable pandemic response. We sought to understand the key activities needed to equip providers to conduct in-home screening, facilitate vaccination, and assist and support families through the COVID-19 pandemic.

**Methods:**

We designed a module to guide Community Health Workers’ (CHWs)’ assessment of families’ knowledge and concerns about COVID-19, provision of education on protective behaviors, recommendations, referrals, and follow-up. The module was delivered to 63 families from January to March 2021. Three action learning community sessions helped explore CHWs’ experiences. Surveys and key informant interviews with CHWs, site leadership, and state public health leaders served to evaluate this strategy.

**Results:**

Study participants considered the module helpful in guiding CHWs’ conversations with families about COVID-19. CHWs stated that they could adapt to using the module to gauge the level of acceptance of public health messages within their communities, connect with families, convey real-time public health messages, and link families and communities to essential services.

**Conclusions:**

We developed a viable module for rapidly engaging community-based agencies and CHWs in a public health emergency response that could be broadly translated and disseminated.

## Introduction

The COVID-19 pandemic highlighted the need for culturally appropriate strategies to support the rapid scale-up of the public health (PH) response. Local communities needed tailored approaches to engage their members in understanding and accepting the changing messages about the pandemic. PH departments alone were unable to effectively reach groups that were disproportionately impacted by the pandemic and associated shutdowns (Brown et al. 2020; Caxaj et al. 2021; Lau Sosa 2022). These communities experienced delays in navigating processes for testing and breakdowns in messaging, contributing to disproportionate rates of disease and other adverse health outcomes. We designed and pilot-tested a culturally appropriate intervention to support a population-level response to the pandemic. Our aims included: 1) engaging community members, local PH, and health care providers in identifying critical activities and policies needed for equitable and effective COVID-19 screening, contact tracing, and vaccination; 2) designing an intervention where Community Health Workers (CHWs) conduct in-home screening, promote and support testing, encourage protective behavior change, assist in contact tracing, and, ultimately, vaccination; and 3) pilot testing the intervention with two community-based agencies using CHW models.

Our process was community-engaged, using the PRECEDE-PROCEED model (Green and Kreuter 1999), which includes partners in all stages, from concept design to evaluation. It also incorporated some of the principles of the Whole Community Approach from the Federal Emergency Management Agency (FEMA) (Sobelson et al. 2015). This project promoted working with existing social structures that support community adaptability and wellbeing. We built on the community’s strengths and adaptability by working with trusted community members to convey the messages and educate them on the procedures. This paper describes our process which demonstrates a viable option to engage vulnerable communities in future public health emergency response.

The proposed approach is novel in six ways: 1) worked with existing community organizations serving historically marginalized populations at higher risk for COVID-19; 2) took a lifespan approach that leveraged the multigenerational involvement of parents and grandparents; 3) integrated across public health, community service organizations, and communities; 4) engaged CHWs as a part of the public health workforce; 5) created a strategy to rapidly increase capacity and model for sustainability; 6) was designed for dissemination and broad implementation.

This project showed that an approach like this can help to overcome the lack of trust in PH and allow people to consider recommended safety/preventive measures, not limited to a pandemic.

## Methods

All community partners (Fig. 1) were engaged starting in the planning phase. We implemented the project with two community-based programs supporting families with young children facing higher poverty rates: one in an urban community and the other in a rural part of the state. The UW-Madison Institutional Review Board determined the project was not research.

### Pilot sites

The two pilot sites vary in staffing, location, and population served. They both employed staff considered CHWs. Each site identified five staff members to implement the intervention with families for three months, from January to March 2021. Site A is located in the largest urban area in Wisconsin. They provide doula and breastfeeding services to pregnant and postpartum families. This agency’s staff are representative of the populations they serve. All participating providers from this site were compensated $500 in cash. Site B is an early childhood home visiting program in a community action program agency in a rural area. The home visiting program staff are early child development providers who serve families from five counties. Site B was federally funded and could not accept compensation.

This project utilized the PRECEDE-PROCEED model (Green and Kreuter 1999), which includes partners in all stages, from concept design to evaluation (Table 1). It also incorporated some of the principles of the Whole Community Approach from the Federal Emergency Management Agency (FEMA) (Sobelson et al. 2015).

### Project design: PRECEDE

From June to December 2020, all partners worked together to identify the desired result of the pilot, set priorities, and better understand predisposing, enabling, and reinforcing factors related to the pandemic response. They also considered administrative and policy factors that were impacting the PH response. Three subgroups were established and met monthly to lead different aspects of the work at this stage. The Training and Support group developed a module (S1) to guide CHW’s conversations with clients, designed CHW training, and established a resource library to support the use of the module. The Linkages group focused on strengthening connections to local PH. The Data & Evaluation group developed an evaluation plan and identified relevant measures. At the end of the PRECEDE phase, the groups designed an intervention incorporating participant training, a module, and a resource library. They planned learning collaborative sessions to support implementation.

### Implementation: PROCEED

The pilot started with 90-min virtual training sessions with participating CHWs and supervisors from each site separately. This CHW training provided background on COVID-19, an overview of the PH system in Wisconsin, and an overview of the project. CHWs were introduced to the module, which would guide their conversations with clients. CHWs were also introduced to an electronic resource library containing resources in English and Spanish on various topics related to COVID-19 curated from reputable PH sources. A menu based on the content was added to the resource library to share with families to allow clients to choose topics they were most interested in discussing. CHWs were given access to the CHW training slides, the module, the resource library, and the menu after the training. As participants requested, we made the module and menu available digitally and in a printable format.

CHWs were asked to use the module to assess clients’ understanding of COVID-19 testing, contact tracing, and vaccination, their own risk of acquiring COVID, and measures they could take to reduce their risk. After the initial assessment, the module guided the CHWs through offering education or reinforcement on five essential protective behaviors (wearing a mask, social distancing, quarantine, isolation, and COVID-19 testing), referrals as needed (accessing additional benefits, testing, trusted sources for information), and follow-up.

We met with the participants and their supervisors during the implementation phase in three Action Learning Community (ALC) sessions. These sessions were held virtually and allowed CHWs to share, with the study team and each other, their experiences using the module with clients. During ALC sessions, we included additional education based on participants’ interests or concerns. For example, multiple participants were very interested in learning more about COVID-19 vaccines. The team arranged for a guest speaker to join the next ALC session to give a presentation about vaccines. The ALC sessions were co-facilitated by a participant from one of the partnering organizations and recorded. Following each ALC session, participants completed an evaluation and were able to request topics for future sessions.

### Evaluation: PROCEED

For the evaluation we used the RE-AIM framework. The goals of the evaluation were jointly developed with partners to include demonstrating the feasibility of incorporating CHWs in the pandemic response, increasing the CHWs’ skills and knowledge around COVID-19, providing an avenue to better inform communities about COVID-19 and hear from communities about what they need and bring it back to PH; and identifying the key components of this intervention (module, CHW training, and the ALC).

The evaluation plan used a mixed-methods approach and collected from each phase of the implementation. Pre- and post-training surveys were collected after each site’s initial training. We conducted thematic analysis of qualitative data from transcripts for each ALC Session generated by virtual platform recordings. Each pilot site supervisor provided aggregate data collected by the community health workers. At the end of the intervention, we completed key informant interviews (S5) with supervisors from each organization to assess their overall experience with the intervention and their intent to maintain the use of the modules.

The original partners were convened and presented the implementation results. The study team then engaged partners in co-designing a toolkit to facilitate others adapting this pilot to their settings.

## Results

As reported by pilot site supervisors the intervention reached 392 individuals (190 adults, 202 children) in 63 households through 146 visits over 3 months (Jan-Mar 2021). While most visits were conducted virtually (109 total), some participants reported conducting in-person visits (37 total). Home visitors visited forty-one households, and doulas visited twenty-two households.

### Module, resource library & menu

Participants documented their discussions and any recommendations, referrals, and follow-ups for each topic area.

Families were engaged in various COVID-19 education topics, which reflected the earlier stages of the pandemic. Isolation, quarantine, and COVID-19 testing were the topics most discussed (Fig. 2). CHWs also assessed individuals for areas of life impacted in 90 visits.

Participants described the module as easy to blend into their established visit structure and not adding too much time to their visits. They were already conversing with families about COVID; the module and the menu allowed them to go into the topics more deeply. The menu allowed families to select topics of interest using icon tiles.

Supervisors ultimately saw how much staff members had learned.

“The level of information our staff got made it worth it. With the times we face now, it was good that they had things to share with people.”

“[Supervisor] was impressed with how much they learned.”

Supervisors also felt the intervention did not add to staff workload. From their perspective, CHWs would have been doing similar work to support COVID-19 even if they had not been participating in the pilot. One supervisor estimated that it took 1–2 h of their own time most weeks to perform tasks related to the intervention, such as helping to plan the intervention, talking with staff about the process, checking in with staff to determine how things were going and address any questions; coordinating personal protective equipment (PPE) for CHWs and their clients; and putting together printed binders containing all of the resources.

While CHWs varied in how they incorporated the module into their existing workflow during a visit, all felt it was a good fit and did not take away from their regular visits.

Most participants were able to get through the entire module during their visits. Even when parts of the module were not completed in one visit, they felt comfortable covering it during future visits. The CHWs were adapting the module to different situations and balancing it with other priorities based on their overall assessment of their client’s needs.

A supervisor from each pilot site participated in a 30–45-min virtual interview to better understand their perspectives on the intervention.

Supervisors agreed that their overall experience with the project was positive. They felt that time was used effectively, that they were included in the planning, and that they received adequate support and communication from the study team throughout.

“From my perspective, it was a positive thing.”

“… at the end of the day, the children and parents will benefit. The more information they have about anything – specifically around their health – is a good thing”.

### Community health worker training

All ten CHWs completed a pre-training survey, and eight of ten CHWs completed the post-training survey. (S2).

All respondents agreed or strongly agreed that the training helped them feel more knowledgeable about PH in general, PH’s role in the pandemic, testing, contact tracing, and prevention. CHWs also found it valuable to learn about the module, resource library, and menu they would use throughout the intervention.

“I liked the resources you had. The website where you could get everything. Especially having them in Spanish too.”

CHWs requested additional information on a vaccine and the impact of COVID on pregnancy and breastfeeding. They praised the training and the level of interaction overall but suggested including relationship-building activities and providing time and space for connecting. Most agreed or strongly agreed that the material was appropriate to their background experience.

Two months after the end of the intervention, eight participants completed a post-intervention survey (S3), which included the same COVID Knowledge questions asked in the pre-and post-training surveys and asked about participants’ experience with different aspects of the intervention. Participant’s knowledge about pregnancy-related COVID topics remained strong, though there was still some confusion about quarantine and isolation.

All respondents agreed the intervention was a good fit and easy to incorporate. Most (7/8) decided to continue using the module.

### Action learning community

CHWs agreed that the ALC sessions were a valuable use of their time and felt comfortable speaking up and contributing. Participants valued the opportunity to hear about others’ experiences, ask questions, build community, and connect with other CHWs to share knowledge and learn from experts/guest speakers.

Supervisors greatly valued the shared information and expertise (i.e., guest speakers).

“Having the speakers come in was cool.”

Participants also brought up their topics for discussion during ALC sessions (Fig. 3). Some topics were based on what they were hearing from families, such as vaccine safety and efficacy by race, protective behaviors, navigating complex isolation situations (e.g., isolating with children at home), child well-visits, breastfeeding for mothers with COVID-19, babywearing and attachment, and housing, income, access to healthy food during a pandemic.

Other discussion topics were CHW’s needs: commiserating around the perceived politicization of COVID-19 and related topics, setting boundaries with clients, worker safety and wellbeing, and how to respond to client resistance or hesitation around specific topics.

Some supervisors shared that staff wished the intervention had been implemented earlier in the pandemic, as by the time the intervention was implemented, families were getting “tired of talking about COVID,” and there was more misinformation for everyone to navigate.

One supervisor shared the importance of this for reaching populations living in rural areas.

“In rural communities, sometimes information from your neighbor is more powerful and impactful than public health officials from higher up. Those informal settings there [via home visits] are more opportunities to discuss issues than formal settings.”

The ALC sessions also allowed participants to foster connections around interests and needs unrelated to the pandemic. In one instance, participants discussed the possibility of collaborating to make an informational video about babywearing for clients. Participants also brainstormed how to better support each other by holding practice sessions to run through potential scenarios they might encounter with clients and finding ways to help each other when visiting families for whom English was not their primary language.

Participants shared important themes regarding their unique approach to their clients.

#### Pausing to listen

Overall, participants discussed their approach as being ‘client-led,’ meaning they would prioritize conversing with clients, meeting them where they were, and “letting conversations flow” rather than asking too many questions. Many participants described their approach as one of transparency and humility, letting clients know they were also learning the content, which gave everyone a sense of going through the experience together. Participants prioritized listening and understanding clients’ concerns so they could tailor their next visit(s) accordingly. They mentioned health literacy and “breaking down” medical terms whilst supporting clients’ education and encouraging them to advocate for themselves with other health providers.

#### Sharing decision-making

Participants felt strongly about not “pushing” issues that families were not interested in, providing unconditional support, and supporting client’s sense of autonomy and choice whilst delivering the best information they could.

“We focus on not making decisions for our client.”

## Discussion

### Main finding

Our results showed that with brief training, community health workers were able to provide essential public health education to families about protective measures which demonstrates that PH departments can effectively engage community-based programs with longstanding relationships with families in emergency responses. In light of the difficulty public health departments had in equitably reaching populations that were heavily impacted by the pandemic health departments should consider this approach in future responses. A systematic approach that includes support for the community-based organizations and CHWs is essential to their capacity to engage in a PH response fully. Our findings indicate that all components (module, training, resource library, menu, and ALC) were needed for the success of the intervention. The two sites viewed the structure of support and guidance as critical to a rapid uptake of these community-based programs.

### What is already known on this topic

The recent pandemic highlighted the need for PH agencies to have stronger connections to communities that were less trusting of governmental PH efforts (Nana-Sinkam et al. 2021; Wong and Kohler 2020). Community-based organizations providing services to local communities can effectively be included in emergency response and be an essential link to populations with negative experiences with PH (Cheng et al. 2020; Regnier-Davies et al. 2022; Roels et al. 2022). Interventions must also be designed to incorporate the unique differences across communities and regions. This need for tailoring further emphasizes the advantage of a PH response focused on community-based programs that directly work with individuals and families.

### What this study adds

This study demonstrates the feasibility of designing a process to engage community-based programs in a PH emergency response. We provide a path, tools, and resources to guide others in adapting and implementing a similar design. Additionally, we worked with partner organizations and CHWs to develop a toolkit [S4] that guides using and adapting the module and associated training, establishing an ALC, and creating a resource library with a corresponding menu of topics. The toolkit can be used by anyone [https://prc.wisc.edu/chw/] interested in bringing culturally appropriate and timely information about critical PH issues to families in their communities.

We also interviewed a PH practitioner working in emergency response who advised the project. They supported the ideas of a toolkit clearly outlining the roles of CHWs, describing examples of situations in which CHWs could be incorporated and solutions to common problems identified.

“What are the roles of CHWs who have experience working with people and understanding the needs of the community and the populations that have been hard for us to reach? How do we tie this all together to reach the populations with people they already trust? It is really [about] building this network.”

They mentioned challenges with emergency funding, most of which is federal funding, and may not be as flexible for use in work like this. There was also concern that funding would only be available in the short term.

### Limitations of this study

While our project demonstrated an effective approach that can be replicated to engage community-based programs in a public health response, there were some limitations that should be considered when interpreting the results. The project was piloted with two organizations and thus the findings are not generalizable. However, the two pilot sites did represent diverse locations and populations that were recognized as marginalized during the pandemic response. Another limitation of this project was that there was a loss of several of the original participating partners. Multiple organizational partners experienced unexpected staffing changes caused by the pandemic and were no longer able to commit to participating, reducing the number of planning partners who remained engaged through to the end. We were also unable to engage local public health of the pilot site communities during the implementation phase. We were successful in involving the state-level preparedness staff in the planning. A local public health nurse from another county, and a state-level home visiting nurse consultant participated in an ALC discussion.

## Conclusion

The COVID-19 pandemic brought to light stark inequities and highlighted limitations of the public health system in our state to effectively reach historically marginalized communities. In this project we demonstrated that working through trusted local home-visiting programs to promote accurate information and protective measures with community members, can support the dissemination of critical public health messages during a crisis period. Future research should continue to explore innovative approaches to connecting local community partners with public health departments, to deliver essential public health information.

## Supplementary Material

Supplementary Material

**Supplementary Information** The online version contains supplementary material available at https://doi.org/10.1007/s10389-025-02615-w.

## Figures and Tables

**Fig. 1:**
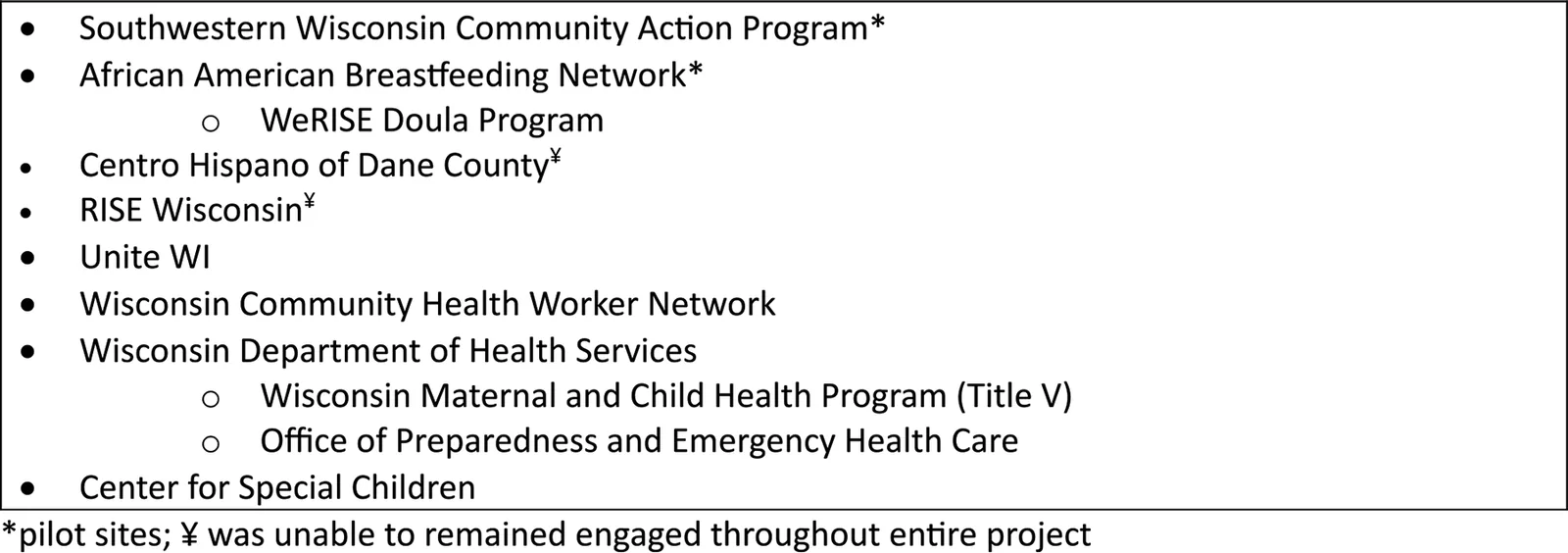
Community Partners

**Fig. 2:**
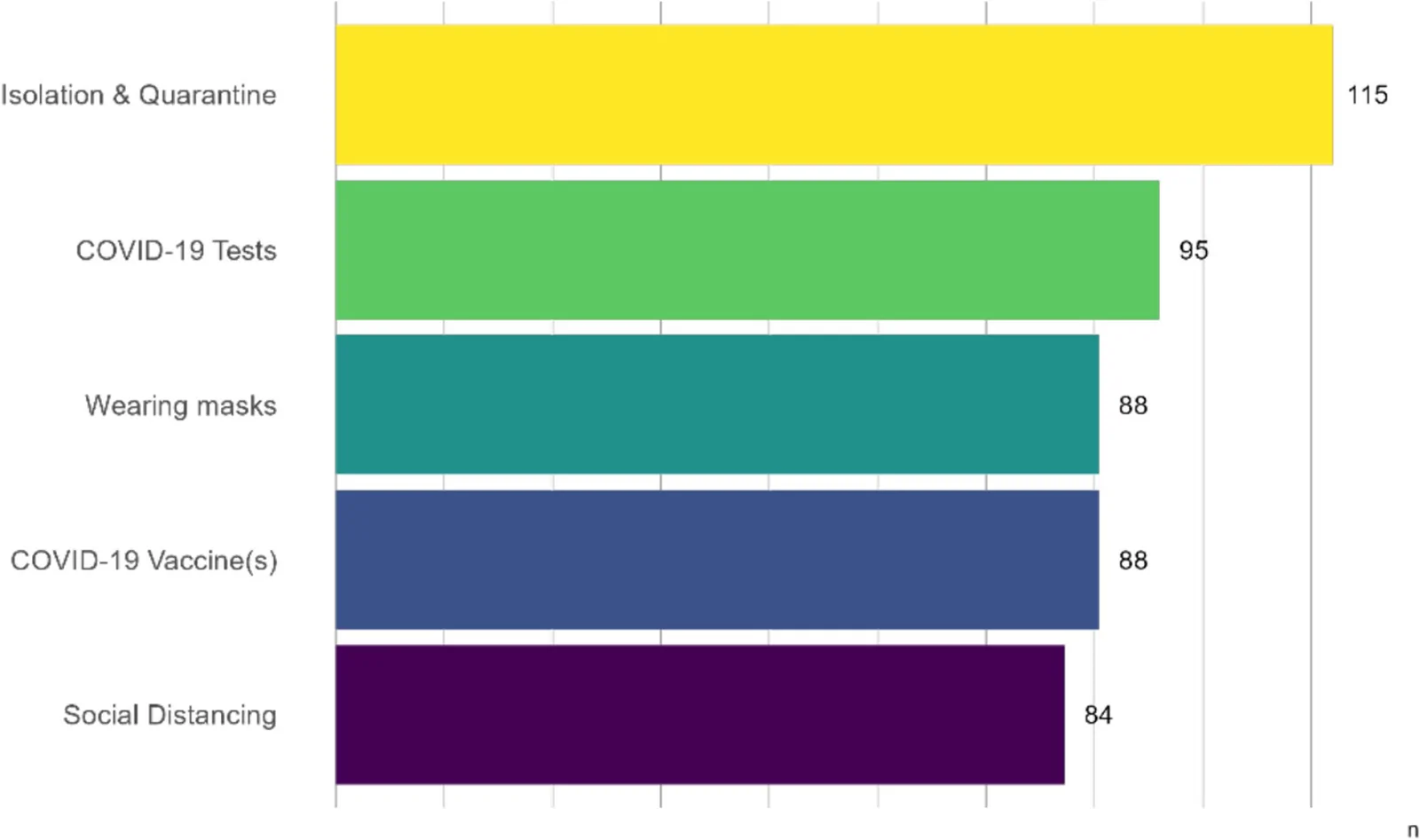
Topics of interest selected by clients

**Fig. 3:**
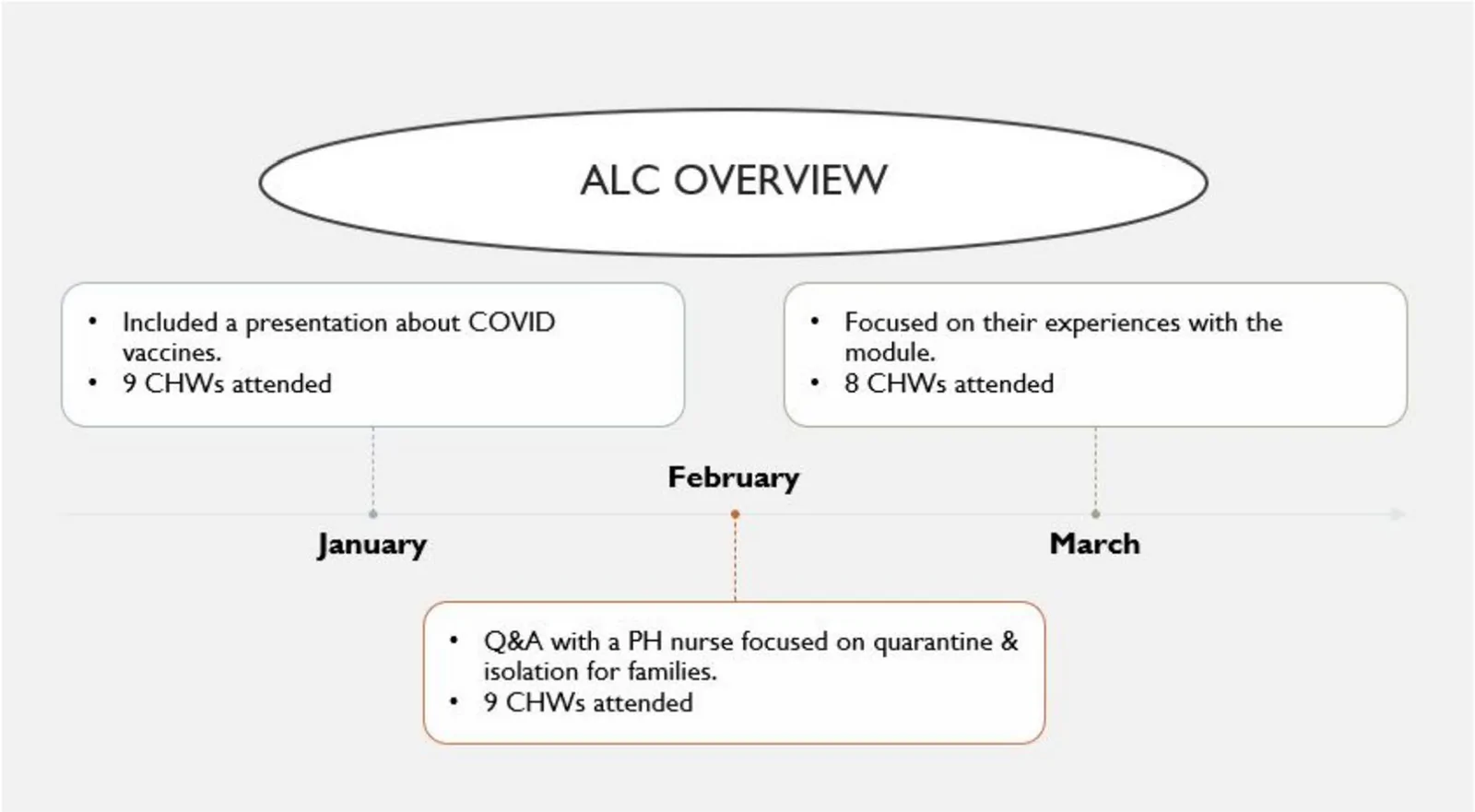
Action Learning Community Sessions

**Table 1:** Overview of Project Implementation Process & Timeline

PRECEDE-PROCEED Planning Model	Aims	Activities	Evaluation Components
PRECEDE Identify the ultimate desired results and set priorities	Engage community members and stakeholders to identify critical activities needed for equitable and effective active COVID-19 case finding, contact tracing, and vaccination, including relevant policies, while considering the predisposing, reinforcing, and enabling contextual factors	Jun. – Aug. 2020 Engaged partners and established three subgroups with partner leads Reviewed existing COVID trainings and available resources Agreed on sections of module: COVID education, risk assessment, recommendations, referrals, follow-up	
PRECEDE Identify predisposing, enabling and reinforcing factors, administrative and policy factors	Design an intervention using CHWs to conduct in-home screening, promote and support testing, encourage protective behavior change, assist in contact tracing, and ultimately navigate to vaccination	Sept. – Dec. 2020 Finalized pilot sites Tailored approach to the unique needs of each community Finalized intervention design (3 major components): Training, Module with resource library, and Action Learning Community	
PROCEED Implementation	Pilot test using two community agencies with community health worker models measuring important process outcomes and generating estimates of impact on health	Jan. 2021 Launched pilot with training session for CHWs and supervisor(s) from each site Jan. – Mar. 2021 CHWs used module, menu and resource library during visits with clients Held three Action Learning Community Sessions (Fig. 3)	Pre- and post-training session surveys Evaluation survey after each ALC session Qualitative data from ALC session transcripts
PROCEED Process, Impact and Outcome Evaluation		Apr. – Jun. 2021 Evaluated pilot using all evaluation components (next column) Reconvened partners to review results and design toolkit Began dissemination of toolkit through partners and on UWPRC website	Aggregate data submitted by supervisors collected via module Key informant interviews with supervisors

## Data Availability

Data is available on request.
