# Supplementary material for: Connecting with community in a public health emergency: a pilot project example for local public health and the communities they serve
Source: J Public Health (Berl). Author manuscript; Available in PMC 2026 Nov 5. (PMC13734717; doi:10.1007/s10389-025-02615-w)
Supplement: Supplementary file 1 [file NIHMS2141574-supplement-Supplementary_Material.docx]

Connecting with community in a public health emergency: a pilot project example for local public health and the communities they serve

*Supplementary Material*

**Supplementary Figure 1**: Building a Public Health Reserve with Community Health Workers Project COVID-19 CHW Module

***Building a Public Health Reserve with Community Health Workers Project***

*COVID-19 CHW Module*

CHW (preferred Name or initials): ­­______________	Client: ________­­­­­______________

#adults in household: _______	#children in household: _______

***Visit 3***	Date: ________

\# present (adults/children): ___/___

❑ Virtual	❑ In-person

***Visit 2***	Date: _______

\# present (adults/children): ___/___

❑ Virtual	❑ In-person

***Visit 1***	Date: _______

\# present (adults/children): ___/___

❑ Virtual	❑ In-person

**Initial Assessment**

- Purpose: to identify the family’s baseline understanding of COVID-19 and protective behaviors.
- Please include all full and partial household members in your assessment.
- Complete full assessment at initial contact; then revisit assessment for changes at visits #2 and #3.

**Assessment: COVID Knowledge**

Example: *“What do you know about COVID-19?”*

**Visit 1:** ❑	**2:** ❑	**3:** ❑

**Notes from Visit 1:**

**Notes from Visit 2:**

**Notes from Visit 3:**

**Assessment: COVID Concerns**

Example: *“What are you most concerned about related to COVID-19?”*

**Visit 1:** ❑	**2:** ❑	**3:** ❑

**Notes from Visit 1:**

**Notes from Visit 2:**

**Notes from Visit 3:**

**COVID Education**

- We encourage you to include all full and partial household members in your discussion.
- At initial contact: Introduce essential topics; offer menu; cover additional topics based on client needs/interests as time allows.
- At visits #2 and #3: revisit essential topics to assess for changes, reinforce/redirect as necessary, offer menu and cover additional topics as needed.
- Space education out over all three visits based on client/household preference.

For your discussion of the essential topics that follow, we encourage you to start by asking a few questions to help determine what your client(s) already know and/or have experienced in relation to the specific topic. Please offer educational materials related to each topic as is most relevant to your client’s needs and experience.

*I’d like to start with some basics and then move into the other topics you select.*

**1. Testing**

Start by discussing client’s experience and knowledge around COVID-19 testing. Below are a few questions that you may want to consider as part of your discussion:

*Has anyone in the household been tested?*

- *How many times?*
- *Where did they go?*
- *What was the type of test?*
- *How easy was it to access?*

Offer educational materials related to testing as is most relevant given your client’s experience & needs.

**COVID-19 Tests**

**Visit 1:** ❑	**2:** ❑	**3:** ❑

**Handout(s) given:**

❑ Types of tests

**Did you…**

❑ Introduce, ❑ Reinforce, or ❑ Redirect

…information/knowledge on this topic?

**Notes from Visit 1:**

**Notes from Visit 2:**

**Notes from Visit 3:**

**2. Isolation & Quarantine**

Start by discussing client’s experience and knowledge around isolation & quarantine.

Below are a few questions that you may want to consider as part of your discussion:

*Has anyone in the household had to isolate or quarantine?*

- *Describe what you did to isolate/quarantine*
- *Did any symptoms develop?*

Offer educational materials related to isolation & quarantine as is most relevant given your client’s experience & needs.

**Isolation & Quarantine**

**Visit 1:** ❑	**2:** ❑	**3:** ❑

**Handout(s) given:**

❑ Isolation & Quarantine

**Did you…**

❑ Introduce, ❑ Reinforce, or ❑ Redirect

…information/knowledge on this topic?

**Notes from Visit 1:**

**Notes from Visit 2:**

**Notes from Visit 3:**

**3. Essential Protective Behaviors**

Start by discussing client’s experience and knowledge around the following protective behaviors:

1. Wearing a mask
2. Social distancing

Ask what protective behaviors they follow and how often (include all household members). Below are a few questions that you may want to consider as part of your discussion:

*What kinds of things are you and your family doing to protect yourselves from COVID? Do you:*

- *wear a face covering outside of your home?*
- *avoid going inside bars and restaurants?*
- *Keep at least 6 feet apart from people who are not members of your household?*
- *Avoid groups of 10 or more persons?*

Offer educational materials related to wearing a mask and social distancing as is most relevant given your client’s experience & needs.

**Wearing a mask**

**Visit 1:** ❑	**2:** ❑	**3:** ❑

**Handout(s) given:**

❑ Masks

**Did you…**

❑ Introduce, ❑ Reinforce, or ❑ Redirect

**…**information/knowledge on this topic?

**Notes from Visit 1:**

**Notes from Visit 2:**

**Notes from Visit 3:**

**Social Distancing**

**Visit 1:** ❑	**2:** ❑	**3:** ❑

**Handout(s) given:**

❑ Social distancing

**Did you…**

❑ Introduce, ❑ Reinforce, or ❑ Redirect

…information/knowledge on this topic?

**Notes from Visit 1:**

**Notes from Visit 2:**

**Notes from Visit 3:**

**4. COVID-19 Vaccine(s)**

- *Have you thought about the getting the COVID-19 vaccine?*
- *What questions or concerns do you (or others in the household) have regarding the COVID-19 vaccine(s)?*

**COVID-19 Vaccine(s)**

**Visit 1:** ❑	**2:** ❑	**3:** ❑

**Notes from Visit 1:**

**Notes from Visit 2:**

**Notes from Visit 3:**

**5. Other Protective Behaviors** *(Optional)*

- Using hand sanitizer/washing hands
- Virtual school
  - If there are any children in the home – *Do any of the children attend a daycare? school in person? Virtually?*
- Limiting going out for local activities
  - *Have you (or anyone in the household) attended any social gatherings or other local activities/events in the last 6 months?*

**COVID Education – Menu**

- Present the menu of education topics to family and ask what they would like more information on.
- Provide links to materials about specific topics or send as attachment to support conversation.
- Invite other household members to also participate or leave materials for family to share with others.
- Circle or mark any items clients or family members are interested in.

*Which would you like to start with first? Let’s plan to go into the rest at our next visit.*

OR

*I will send you some more information and we can talk about it at our next visit*

❑❑❑❑

❑❑❑❑

❑❑❑❑

❑❑❑❑

**COVID Education - Notes from menu conversation**

**Notes from Visit 1:**

**Notes from Visit 2:**

**Notes from Visit 3:**

**Areas of Life Impacted by COVID**

- Discuss recommendations to maintain family wellness and direct families to local resources using your agency list of local providers to support recommendations.
- Include detailed information on how to access the resources per agency practices.
- Follow agency protocols for emergencies.
- For initial visit:
  - Will vary based on family’s knowledge and circumstances related to COVID-19.
  - Basic recommendations will be introduced at first visit.
- For follow-up:
  - Follow up on all recommendations and referrals per agency practice.
  - Reinforce the basic recommendations at visits 2&3.
  - New issues will be addressed if requested/needed at visits 2&3.
- Ask if they were able to follow through on the recommendations and/or referrals.
- If not, ask about the barriers they encountered.
  - Offer assistance with troubleshooting the identified barriers within capacity of CHW.

NOTE: The assessment table contains a list of topics that might come up in these conversations. For any topics that come up that you would like to take more notes on, please use the boxes on the following pages.

| **Assessment:** Ask how the family has been impacted | | | |
| --- | --- | --- | --- |
| **Example**: *How are you coping during these difficult times?* | **Visit 1** | **Visit 2** | **Visit 3** |
| Working outside the home |  |  |  |
| Childcare needs |  |  |  |
| Health risks |  |  |  |
| Job loss |  |  |  |
| Concerns about safety |  |  |  |
| Economic and food insecurity |  |  |  |
| Accessing healthcare - Going for preventive routine care |  |  |  |
| Accessing healthcare - Comfort in using urgent/emergent care |  |  |  |
| Accessing mental health care |  |  |  |
| Ability to care for yourself and family (including family living separately) |  |  |  |
| Difficulty caring for pets |  |  |  |
| [other topics] |  |  |  |

**Topic:**

**Visit 1:** ❑	**2:** ❑	**3:** ❑

**Referrals:**

**Recommendations:**

**Notes from initial conversation:**

**Follow-up at next visit:**

[Document if client successfully followed through on referral]

**Optional additional follow-up:**

**Topic:**

**Visit 1:** ❑	**2:** ❑	**3:** ❑

**Referrals:**

**Recommendations:**

**Notes from initial conversation:**

**Follow-up at next visit:**

[Document if client successfully followed through on referral]

**Optional additional follow-up:**

**Topic:**

**Visit 1:** ❑	**2:** ❑	**3:** ❑

**Referrals:**

**Recommendations:**

**Notes from initial conversation:**

**Follow-up at next visit:**

[Document if client successfully followed through on referral]

**Optional additional follow-up:**

**Topic:**

**Visit 1:** ❑	**2:** ❑	**3:** ❑

**Referrals:**

**Recommendations:**

**Notes from initial conversation:**

**Follow-up at next visit:**

[Document if client successfully followed through on referral]

**Optional additional follow-up:**

**Topic:**

**Visit 1:** ❑	**2:** ❑	**3:** ❑

**Referrals:**

**Recommendations:**

**Notes from initial conversation:**

**Follow-up at next visit:**

[Document if client successfully followed through on referral]

**Optional additional follow-up:**

**Topic:**

**Visit 1:** ❑	**2:** ❑	**3:** ❑

**Referrals:**

**Recommendations:**

**Notes from initial conversation:**

**Follow-up at next visit:**

[Document if client successfully followed through on referral]

**Optional additional follow-up:**

**Supplementary Figure 2**: *Pre & Post-Training Surveys*

*COVID-19 Pre-Training Survey*

**ID #**: _____

**Have you attended any trainings or educational lectures about COVID-19 in the last 6 months?**

- Yes
- No
- I don’t remember

**Have you attended any trainings or educational lectures about public health and/or emergency response in the last 6 months?**

- Yes
- No
- I don’t remember

**Public Health & Emergency Response**

**Instructions**: Please select your level of agreement with each of the following statements.

|  | Strongly Disagree | Disagree | Neutral | Agree | Strongly Agree | N/A |
| --- | --- | --- | --- | --- | --- | --- |
| I understand the role of state and local public health organizations in control and prevention of communicable disease in general. |  |  |  |  |  |  |
| I understand the role of state and local public health organizations in responding to a pandemic like COVID-19. |  |  |  |  |  |  |
| I feel well informed about what my local public health department is doing to respond to COVID-19. |  |  |  |  |  |  |
| I know where to go to find current public health policies/ regulations related to COVID-19. |  |  |  |  |  |  |
| I know who my local public health representatives are and how to contact them. |  |  |  |  |  |  |
| I feel comfortable connecting with my local public health representatives in relation to my work. |  |  |  |  |  |  |
| When I have collaborated with public health representatives in the past in relation to my work, the experience has been positive. |  |  |  |  |  |  |
| I see my work as a key component of the public health response to COVID-19. |  |  |  |  |  |  |
| I feel that *others* see my work as a key component of the public health response to COVID-19. |  |  |  |  |  |  |

**I collaborate with local public health in carrying out my work**

- Never
- Rarely
- Sometimes
- Often
- This does not apply to my work

**COVID-19 Self-Assessment**

**Instructions**: Please select your level of agreement with each of the following statements.

|  | Strongly Disagree | Disagree | Neutral | Agree | Strongly Agree | N/A |
| --- | --- | --- | --- | --- | --- | --- |
| I feel prepared to talk with the clients/families I work with about: |  | | | | | |
| - Testing |  |  |  |  |  |  |
| - Vaccination |  |  |  |  |  |  |
| - Contact Tracing |  |  |  |  |  |  |
| - Prevention |  |  |  |  |  |  |
| I feel prepared to assess patients for risk factors for severe illness from COVID-19? |  |  |  |  |  |  |
| I am competent in addressing most of the needs of the clients/families I work with related to COVID-19 |  |  |  |  |  |  |

**COVID-19 Knowledge Questions**

The following questions test your knowledge about COVID-19. Please select the most correct answer to the best of your knowledge.

***True or False***

The virus that causes COVID-19 is thought to spread mainly from person to person through respiratory droplets produced when an infected person coughs, sneezes, or talks.

Someone could feel perfectly healthy and still have COVID-19 and transmit the virus to others.

Adults who are 65 years or older and people of any age with underlying medical conditions are at higher risk of becoming severely ill from COVID-19.

Pregnant people are at an increased risk for severe illness from COVID-19 and may be at increased risk for complications such as preterm birth.

Women with confirmed or suspected COVID-19 can breastfeed if they wish to do so and breastfeeding should still be encouraged and supported.

***Multiple Choice***

Once a person is exposed, how long before they might experience symptoms?

a. 12-24 hours

b. 30-60 days

c. 2-14 days

d. 3-4 weeks

Which test type shows if you have been infected with coronavirus in the past?

a. Molecular test

b. PCR test

c. Antibody test

A negative antigen test means that you do not have an active coronavirus infection. True or False

Which test is more likely to miss an active infection of coronavirus?

a. Molecular (PCR) test

b. Antigen test

c. Antibody test

Which test type can tell you if you are infected with coronavirus now?

a. PCR test

b. Antibody test

c. Urine culture

All of the following are things you can do to protect yourself from COVID-19, *except*:

a. wear a mask that covers your nose and mouth

b. stay at least 6 feet apart from people you don’t live with

c. avoid opening your mail

d. wash your hands often with soap and water

***Isolation vs. Quarantine - Fill in the Blank***

Please fill in the blank with the correct term:

____	____ keeps someone who is sick with the virus away from others, even in their own home.

____	____ keeps someone who might have been exposed to the virus away from others.

**General Questions**

***Which of the following best describes your current or most recent position?**

- Community Health Worker
- Promotor(a)
- Community Health Representative
- Home visitor
- Doula
- Other:__________

* **Which best describes the community where you do most of your work?**

- A large city
- A suburb near a large city
- A small city or town
- A rural area

***Since the COVID-19 pandemic response began, where are you doing most of your work? Select up to two (2) options.**

- Hospital
- Nursing home or long-term care facility
- Clinic or outpatient medical facility
- Health department
- Client’s homes
- Office setting
- Working remotely from home
- Out in the community/on the street
- Schools
- Community Centers
- Shelters
- Work Sites
- Religious Centers
- Early childhood education/Head Start
- Other:__________

***In general (not during COVID) what setting do you do most of your work? (e.g. homes, clinics, etc.)**

- Homes
- Clinics/Hospitals
- Community Centers
- Schools
- Work Sites
- Religious Centers
- Shelters
- Street
- Other, specify: ___________________________________________________.

***What kind of training did you receive when you began your current job? (select all that apply)**

- None
- Public Health Issues (AIDS, Cancer, Domestic Violence, etc.)
- Health Education Methods
- Counseling/Mentoring Techniques
- Making Referrals
- Cultural Competency/Health Issues
- First Aid/CPR H Safety
- Leadership Training
- Management/Organizing Skills
- Fundraising/Grant Writing
- Other, specify: ___________________________________________________.

***How many years of experience do you have working with families in the community?** _______________ [*open ended question*]

*COVID-19 Post-Training Survey*

**ID#**: _____

Please list the three most important/valuable things you learned during today’s training.

1. ___________________________
2. __________________________
3. __________________________

**COVID-19 Knowledge Questions**

The following questions test your knowledge about COVID-19. Please select the most correct answer to the best of your knowledge.

***True or False***

The virus that causes COVID-19 is thought to spread mainly from person to person through respiratory droplets produced when an infected person coughs, sneezes, or talks.

Someone could feel perfectly healthy and still have COVID-19 and transmit the virus to others.

Adults who are 65 years or older and people of any age with underlying medical conditions are at higher risk of becoming severely ill from COVID-19.

Pregnant people are at an increased risk for severe illness from COVID-19 and may be at increased risk for complications such as preterm birth.

Women with confirmed or suspected COVID-19 can breastfeed if they wish to do so and breastfeeding should still be encouraged and supported.

***Multiple Choice***

Once a person is exposed, how long before they might experience symptoms?

a. 12-24 hours

b. 30-60 days

c. 2-14 days

d. 3-4 weeks

Which test type shows if you have been infected with coronavirus in the past?

a. Molecular test

b. Antigen test

c. Antibody test

Which test type can tell you if you are infected with coronavirus now?

a. Antigen test

b. Antibody test

c. Urine culture

All of the following are things you can do to protect yourself from COVID-19, *except*:

a. wear a mask that covers your nose and mouth

b. stay at least 6 feet apart from people you don’t live with

c. avoid opening your mail

d. wash your hands often with soap and water

***Isolation vs. Quarantine - Fill in the Blank***

Please fill in the blank with the correct term:

____	____ keeps someone who is sick with the virus away from others, even in their own home.

____	____ keeps someone who might have been exposed to the virus away from others.

***What additional training topics related to COVID-19 would be most helpful to you as a CHW working with families in the community?** __________ *[open ended question]*

**Training Specific Questions**

**Instructions**: Please select your level of agreement with each of the following statements:

|  | Strongly Disagree | Disagree | Neutral | Agree | Strongly Agree | N/A |
| --- | --- | --- | --- | --- | --- | --- |
| The topics covered during this training were relevant to me/my work |  |  |  |  |  |  |
| I obtained knowledge and skills that were consistent with the stated learning objectives |  |  |  |  |  |  |
| I found the content well organized and easy to follow |  |  |  |  |  |  |
| I found the level of detail of the material appropriate to my background and experience |  |  |  |  |  |  |
| The pace of the training was acceptable |  |  |  |  |  |  |
| The information provided during this training was credible |  |  |  |  |  |  |
| The level of participation and interaction was throughout the training was acceptable |  |  |  |  |  |  |
| The trainer(s) seemed knowledgeable and proficient |  |  |  |  |  |  |
| The trainer(s) provided relevant examples during the training |  |  |  |  |  |  |
| I believe I will be able to apply what I learned today in my role as a CHW/CHW supervisor/PH practitioner working with families |  |  |  |  |  |  |
| The information provided during this training will change how I do my work |  |  |  |  |  |  |
| The information provided during this training has helped me feel more knowledgeable about: |  | | | | | |
| - The public health system in general |  |  |  |  |  |  |
| - The public health pandemic response |  |  |  |  |  |  |
| - COVID-19 |  |  |  |  |  |  |
| The information provided during this training will help me better care for clients/families I work with |  |  |  |  |  |  |

***What additional training topics related to COVID-19 would be most helpful to you as a CHW working with families in the community?** __________ *[open ended question]*

Please take a moment to tell us how you think we can strengthen or improve this training. *[open ended question]* ________________________________

**Supplementary Figure 3**: *Post-Intervention Survey*

Thank you for your participation in this pilot! As we work to make the pieces of this intervention available to be used by more organizations, we hope you will take some time to help us understand how the pilot worked for you. Please know that all feedback you share – whether positive or negative – is so important, because it will help us make this as good as possible for other community health workers to use in the future.

**Q1**: Please indicate which organization you work with:

*African American Breastfeeding Network*

*Southwest Wisconsin CAP*

**Section 1: COVID-19 Knowledge Questions**

First, as part of evaluating the intervention, we want to ask you a few brief questions about COVID-19.

**Q2:** Pregnant people are at an increased risk for severe illness from COVID-19 and may be at increased risk of complications such as preterm birth.

*True*

*False*

**Q3:** Postpartum people with confirmed or suspected COVID-19 can breastfeed if they wish to do so and breastfeeding should still be encouraged and supported.

*True*

*False*

**Q4:** ____________ keeps someone who is sick with the virus away from others, even in their own home.

*Quarantine*

*Isolation*

**Q5:** _____________ keeps someone who might have been exposed to the virus away from others.

*Quarantine*

*Isolation*

**Section 2: General Questions**

The following questions are about the intervention overall, including the training, the module, the resource library, and the Action Learning Community.

**Q6: Instructions**: Please select your level of agreement with each of the following statements:

|  | Strongly Disagree | Disagree | Neutral/ Undecided | Agree | Strongly Agree | N/A |
| --- | --- | --- | --- | --- | --- | --- |
| **A:** The COVID-19 module was a good fit with my existing work with families |  |  |  |  |  |  |
| **B:** It was easy/simple to add the COVID-19 module to my existing work with families |  |  |  |  |  |  |
| **C:** If available, I plan to continue to utilize the module in my work with clients. |  |  |  |  |  |  |
| **D:** If it were an option, I would like to continue to participate in an Action Learning Community like the one developed for this project |  |  |  |  |  |  |
| **E:** The training that was provided was enough to prepare me to use the COVID-19 module with families |  |  |  |  |  |  |
| **F:** If available, I would like the training to be regularly updated and offered in the future |  |  |  |  |  |  |

**Q7: Instructions**: Please select your level of agreement with each of the following statements:

|  | Strongly Disagree | Disagree | Neutral/ Undecided | Agree | Strongly Agree | N/A |
| --- | --- | --- | --- | --- | --- | --- |
| **A:** I feel the following component(s) of the intervention served to increase my knowledge and/or skills around COVID-19 |  | | | | | |
| Module (including Menu) |  |  |  |  |  |  |
| Resource Library in Box |  |  |  |  |  |  |
| Training |  |  |  |  |  |  |
| Action Learning Community |  |  |  |  |  |  |
| **B:** I feel the following component(s) of the intervention significantly impacted my ability to guide changes in behavior for my clients related to COVID-19 |  | | | | | |
| Module (including Menu) |  |  |  |  |  |  |
| Resource Library in Box |  |  |  |  |  |  |
| Training |  |  |  |  |  |  |
| Action Learning Community |  |  |  |  |  |  |
| **C:** I feel the following component(s) of the intervention did **not** add value to the intervention or could be eliminated |  | | | | | |
| Module (including Menu) |  |  |  |  |  |  |
| Resource Library in Box |  |  |  |  |  |  |
| Training |  |  |  |  |  |  |
| Action Learning Community |  |  |  |  |  |  |

**Q8: Instructions**: Please select your level of agreement with each of the following statements:

|  | Strongly Disagree | Disagree | Neutral/ Undecided | Agree | Strongly Agree | N/A |
| --- | --- | --- | --- | --- | --- | --- |
| **A:** I feel confident in my ability to discuss *vaccination* with the families I work with in a culturally appropriate way |  |  |  |  |  |  |
| **B:** I feel confident in my ability to discuss *testing* with the families I work with in a culturally appropriate way |  |  |  |  |  |  |
| **C:** I feel confident in my ability to discuss *prevention* with the families I work with in a culturally appropriate way |  |  |  |  |  |  |
| **D:** I feel confident in my ability to discuss *contact tracing* with the families I work with in a culturally appropriate way |  |  |  |  |  |  |

**Section 3: Module, Menu & Resource Library**

The following questions relate to the module, the menu, and the resource library (in Box).

**Q9:** Where did you usually insert the COVID-19 module into your regular visit/discussion with families?

*Beginning*

*Middle*

*End*

*Other: _______*

**Q10:** Were there ever topics/elements of the COVID-19 module that you were not able to get to during your visits?

*Yes*

*No*

*Please Explain:*

**Q11:** Were there topics you normally cover during your visits that suffered/were lost in order for you to fit the COVID-19 module in?

*Yes*

*No*

*Please Explain:*

**Q12:** Were there specific elements of the module that you used most often or found most valuable to support your COVID-19 discussions with families?

*Please Explain:*

**Q13:** Were there specific elements of the module that you used least often or found least valuable to support your COVID-19 discussions with families?

*Please Explain:*

**Q14:** Were there any questions/needs related to COVID-19 that you could not address?

*Yes*

*No*

*Please Explain:*

**Q15:** Did you use the fillable PDF, the printed PDF, or a mix of both?

*Fillable PDF*

*Printed PDF*

*Both*

**Q16:** Did you ever run out of room for notes in the module?

*Yes*

*No*

*Please Explain:*

**Q17:** Did you use the menu with your clients?

*Yes*

*No*

*Please Explain:*

**Q18:** Did you share resources (e.g., printed infographics) from the Resources Library when your clients?

*Yes*

*No*

**Q19:** If you answered “yes” above, did you ever provide resources to your clients that you thought might be shared with people other than your clients (e.g., family members living inside the home, or others living outside the home)?

*Yes*

*No*

*I’m not sure*

*Please Explain:*

**Q20:** Is there anything you would tell other CHWs who were going to be using the module?

___________________________

**Q21:** If you could make changes to the module, what changes would you make?

___________________________

**Q22:** Please use this space to share any other thoughts or suggestions you have regarding the module.

___________________________

**Section 4: Action Learning Community (ALC)**

The following questions relate to the Action Learning Community.

**Q23: Instructions**: Please select your level of agreement with each of the following statements:

|  | Strongly Disagree | Disagree | Neutral | Agree | Strongly Agree | N/A |
| --- | --- | --- | --- | --- | --- | --- |
| **A:** I was satisfied with the way the ALC sessions were structured/run |  |  |  |  |  |  |
| **B:** I had ample opportunity to ask questions and receive answers to my questions during the ALC sessions |  |  |  |  |  |  |

Please take a moment to expand on any of your answers to the questions in the table above. ___________________________

**Q24:** What do you think are the most important parts of an ALC? Please select all that apply.

*Building community*

*Learning from experts*

*Meeting other community health workers*

*Sharing knowledge with others*

*Hearing about others’ experiences*

*Being able to ask and receive answers to questions*

*Something else*

*Please Explain:*

**Q25:** We want to understand who you feel should be members of an Action Learning Community. For each role below, please indicate whether you feel they are essential to the community, they are optional to be part of the community, or if they do not belong in the community.

|  | **Essential** | **Optional** | **Don’t Belong** |
| --- | --- | --- | --- |
| Community Health Workers (CHWs) |  |  |  |
| CHW supervisors |  |  |  |
| Community Leaders |  |  |  |
| Members of a project team |  |  |  |
| Public Health Representatives |  |  |  |
| Others (please explain below) |  |  |  |

*Please Explain:*

**Q26:** What do you think the ideal frequency of an ALC would be?

*Every week*

*Every other week*

*Every month*

*Every other month*

*Something else*

*Please Explain:*

**Q27:** The ALC sessions were 90 minutes long. Do you think that length was:

*Too short*

*Too long*

*Just right*

*Please Explain:*

**Q28:** What, if anything, could have made the ALC sessions feel more welcoming? Ideas related to ALC sessions that meet virtually or in person are welcome.

___________________________

**Q29:** What, if anything, could have made the ALC sessions feel more useful?

___________________________

**Q30:** Are there any suggestions you would give to a facilitator for future ALCs?

___________________________

**Q31:** Is there anything you would tell other CHWs who were going to participate in an ALC?

___________________________

**Q32:** Please use this space to share any other thoughts or suggestions you have regarding the Action Learning Community.

___________________________

Thank you again for participating in this pilot, and for sharing your experiences with us. We could not do this important work without you. Please use the space below to share any last comments, questions, or feedback for us. When you are ready, please press “submit”.

**Q33:** Please share any additional comments here:

___________________________

**Supplementary Figure 4**: Connecting with Community in a Public Health Emergency Toolkit, Toolkit Guide & Summary

The toolkit is located on the UWPRC website, [www.prc.wisc.edu/chw](http://www.prc.wisc.edu/chw).

Toolkit components include:

Module

The module was designed with and for CHWs that work with families in families’ homes. It will guide the CHW through conversations with families to assess their knowledge, provide education, and navigate resources.

Training on Module

A training using PowerPoint slides, provides an orientation for the CHWs on how to use the module and contains the following sections:

• Introduction

• Public Health process

• Understanding COVID-19

• Module design and process

• Action Learning Community (ALC)

Action Learning Community

The Action Learning Community brings the CHWs together to share their experiences with using the module and to learn from each other and guest presenters.

Resource Library

The resource library includes infographics and documents related to COVID-19. All the resources are in English; resources are in Spanish whenever they were available. There is an accompanying menu of topics for clients to select resources for further discussion.

There is also a [Toolkit Guide](https://prc.wisc.edu/wp-content/uploads/sites/1127/2021/08/Toolkit-Guide-Document_FINAL.pdf) – *Connecting with Community in a Public Health Emergency*. For each of the components of the intervention (module, resource library, menu of topics, training, and Action Learning Community), we provide an overview of the component, suggestions for implementation based on our experiences in the pilot, and information to help you customize the component for your own needs. The suggestions we share are based on the experiences from the CHWs – doulas and early childhood home visitors – who piloted this intervention, and their supervisors.

[Toolkit Summary](https://prc.wisc.edu/wp-content/uploads/sites/1127/2021/07/One-Page-Toolkit-Summary.pdf)

**Supplementary Figure 5**: Key Informant Interview Guide

**Interview Guide**

**Key Informant Interviews with Supervisors**

1. What went well for you as a supervisor? (training, module, ALC)
2. Was there anything that could have gone more smoothly for you as a supervisor? (training, module, ALC)
3. As a supervisor what thoughts do you have about the ALCs / your CHWs’ participation?
   a. Are there ways we could improve the ALCs that you think would improve the experience for your CHWs?
4. What would you want other supervisors to know who might be implementing this in the future? (training, module, ALC)
5. Are there ways that we can support supervisors – different from supporting participants – that we did or did not provide?
6. How much time/effort did it take / add to your work to implement this?
   a. What could make it take less time/effort?
   b. How feasible did it feel to do this?
7. Are there any issues or questions you heard from your CHWs that you haven’t shared with us already that you want to share now?
8. How would you describe the work that you did with this intervention, specifically the work you did as a supervisor?
9. Was there anything throughout the intervention that surprised you, either in a good or a bad way?
